# Blockade of the PD-1 axis alone is not sufficient to activate HIV-1 virion production from CD4^+^ T cells of individuals on suppressive ART

**DOI:** 10.1371/journal.pone.0211112

**Published:** 2019-01-25

**Authors:** John K. Bui, Joshua C. Cyktor, Elizabeth Fyne, Shalyn Campellone, Stephen W. Mason, John W. Mellors

**Affiliations:** 1 Division of Infectious Diseases, Department of Medicine, University of Pittsburgh, Pittsburgh, PA, United States of America; 2 Howard Hughes Medical Research Fellows Program, Howard Hughes Medical Institute, Bethesda, MD, United States of America; 3 Discovery Virology, Bristol-Myers Squibb, Wallingford, CT, United States of America; University Hospital Zurich, SWITZERLAND

## Abstract

Blockade of the programmed cell death protein/ligand 1 (PD-1/PD-L1) pathway with monoclonal antibodies (mAb) is now commonly used for cancer immunotherapy and has therapeutic potential in chronic viral infections including HIV-1. PD-1/PD-L1 blockade could augment HIV-1-specific immune responses and reverse HIV-1 latency, but the latter effect has not been clearly shown. We tested the ability of the human anti-PD-L1 mAb BMS-936559 and the human anti-PD-1 mAb nivolumab to increase HIV-1 virion production *ex vivo* from different peripheral blood mononuclear cell populations obtained from donors on suppressive antiretroviral therapy (ART). Fresh peripheral blood mononuclear cells (PBMC), CD8-depleted PBMC, total CD4^+^ T cells, and resting CD4^+^ T cells were purified from whole blood of HIV-1-infected donors and cultured in varying concentrations of BMS-936559 (20, 5, or 1.25μg/mL) or nivolumab (5 or 1.25μg/mL), with or without anti-CD3/CD28 stimulatory antibodies. Culture supernatants were assayed for virion HIV-1 RNA by qRT-PCR. *Ex vivo* exposure to BMS-936559 or nivolumab, with or without anti-CD3/CD28 stimulation, did not consistently increase HIV-1 virion production from blood mononuclear cell populations. Modest (2-fold) increases in virus production were observed in a subset of donors and in some cell types but were not reproducible in longitudinal samples. Cell surface expression of PD-1 and PD-L1 were not associated with changes in virus production. *Ex vivo* blockade of the PD-1 axis alone has limited effects on HIV-1 latency.

## Introduction

Antiretroviral therapy (ART) does not cure HIV-1 infection because of a persistent reservoir of cells carrying intact proviruses that are capable of infectious virus production, leading to virus replication, spread and rebound viremia if ART is stopped [1–8]. The “shock and kill” strategy for an HIV-1 cure aims to deplete the HIV-1 reservoir by reversing latency and promoting the death of infected cells, either by viral cytopathic effect or by immune-mediated killing [9]. Immune checkpoint blockade is a strategy that has been investigated for its potential to enhance HIV-1-specific immunity [10], and promote proviral expression (i.e., provide a “kick”) by activation of infected CD4^+^ T cells. Generally, immune checkpoints regulate the immune system to promote self-tolerance and limit inflammation to minimize collateral tissue damage [10,11]. In chronic HIV-1 infection, immune checkpoint expression is increased both in individuals with uncontrolled viremia and in those on ART with suppression of viremia [12,13], and is associated with more rapid HIV-1 disease progression [14] and shorter time to viral rebound following ART cessation [15]. This important role of immune checkpoint expression is further supported by *ex vivo* studies using human cells and *in vivo* studies in both animal models and humans demonstrating that HIV-1-specific immune function is augmented by blockade of immune checkpoints [10,16–19].

The FDA has approved monoclonal antibodies (mAb) for the treatment of cancer that block the immune checkpoints cytotoxic T-lymphocyte-associated protein 4 (CTLA-4) and the programmed cell death protein 1 (PD-1), or one of its two natural ligands, PD-L1. Ipilimumab, an anti-CTLA-4 mAb, was FDA approved in 2011 for treatment of metastatic melanoma after it was shown to improve overall survival [20]. However, the associated toxicities with ipilimumab are likely too severe to be used in HIV-1-infected individuals on ART who are otherwise healthy [10,21].

Multiple therapies targeting the PD-1/PD-L1 axis have been approved by the FDA. Nivolumab, an anti-PD-1 mAb, is approved for treatment of head and neck squamous cell cancer (HNSCC), Hodgkin lymphoma, advanced melanoma, non-small cell lung cancer (NSCLC), renal cell cancer, and urothelial cancer. Other antibodies targeting the PD-1/PD-L1 axis include pembrolizumab (anti-PD-1), durvalumab (anti-PD-L1), avelumab (anti-PD-L1), and atezolizumab (anti-PD-L1). PD-1 blockade has led to improved survival and lower rates of high-grade toxicities in advanced melanoma compared to CTLA-4 blockade [22,23]. Limited case reports suggest that blockade of the PD-1/PD-L1 axis may also promote anti-HIV immune responses in HIV-1 infected patients [18,24–26]. PD-L1 blockade with BMS-936559 was also shown to be generally well-tolerated in HIV-1-infected participants and resulted in increased HIV-1 gag-specific polyfunctional CD8^+^ T cell responses [16]. These preliminary data suggest that blockade of the PD-1 axis has a potential role in immune control of HIV-1.

To address the possibility that immune checkpoint blockade can reverse HIV-1 latency, the effects of anti-PD-L1 (BMS-936559) or anti-PD-1 (nivolumab) mAb on virus production were assessed *ex vivo* with different cell populations from HIV-1-infected individuals on long-term suppressive ART. Cultured cells were incubated with increasing concentrations of BMS-936559 or nivolumab, with or without anti-CD3/CD28 stimulation, followed by measurement of virion release into the cell culture supernatants. Cell viability and PD-1/PD-L1 expression were also measured and examined for associations with HIV-1 production.

## Results

### Donor characteristics

Experiments were performed with blood samples drawn from 16 chronically HIV-1-infected volunteers on suppressive ART with plasma HIV-1 RNA <50 copies (cp)/mL (Table 1). A subset of donors had samples drawn at multiple, longitudinal time points to assess the reproducibility of observed *ex vivo* responses.

**Table 1:** Donor characteristics.

Donor	Race	Sex	Age	Duration of Infection (est years)	Duration of Viremia Suppression (years)	Nadir CD4^+^ T Cell Count (cells/mm^3^)	Current CD4^+^ T Cell Count (cells/mm^3^)	Plasma HIV-1 RNA (cp/mL by Single Copy Assay)	HIV-1 DNA (cp/10^6^ PBMC)
**O5**	AA	F	59	16	15	13	930	0.7	62
**E1**	CA	M	46	5	4	355	634	10.7	205
**K4**	CA	M	47	19	6	212	980	< 0.4	150
**W1**	CA	M	56	13	7	93	1210	1.3	594
**B3**	AA	F	56	23–25	12–14	410	1373–1505	1.0–1.7	761
**W7**	AA	F	52	18–20	2–4	435	792–1042	1.7–10	529
**D1**	AA	M	52	15–17	6–8	18	412–564	< 0.6–8.5	473
**E4**	CA	F	47	20–22	3–5	272	461–507	< 0.7	133
**E5**	AA	F	61	15–17	8–10	512	784–1033	< 0.6 - < 0.7	793
**T1**	CA	M	61	19–21	13–15	88	630–650	14	144
**A3**	AA	M	42	6	1	323	605	2	513
**C5**	CA	M	40	6–8	6–7	185	699–805	< 0.6	26
**F6**	AA	M	59	21	17	314	1023	15.8	849
**K5**	CA	M	60	24–26	9–10	80	410–759	2–3.8	310
**M1**	AA	M	42	19	1	210	524	10	448
**R1**	AA	M	60	17	16	576	898	< 0.7	36
**Medians**			**54**	**16.5**	**6.5**	**242**	**824**	**1.68**	**379**

Rows in white, dark grey, or light grey are donors whose cells were used in experiments with nivolumab, BMS-936559, or both, respectively. Ranges of values are given for donors on whom longitudinal sampling was performed. AA, African American; CA, Caucasian American; M, male; F, female.

### Blockade with anti-PD-L1 mAb (BMS-936559)

The effects of BMS-936559, a fully human IgG_4_ anti-PD-L1 monoclonal antibody [27], on HIV-1 production were determined for PBMC, total CD4^+^ T cells, and/or resting CD4^+^ T cells, all derived from fresh whole blood. Resting CD4^+^ T cells were CD69^neg^CD25^neg^HLA-DR^neg^. Cells were incubated with either (1) BMS-936559 at concentrations that reflect therapeutic plasma concentrations (1.25, 5, and 20μg/mL) [27], (2) an isotype control antibody, (3) media alone (no-Ab control), or (4) anti-CD3/CD28 antibody-coated beads (Dynabeads, Invitrogen). Following seven days of culture, virion production was measured as HIV-1 RNA in cell-free supernatant using qRT-PCR [28]. Cell viability was measured using CellTiter-Fluor (Promega).

Experiments with BMS-936559 were performed with cells from 12 donors (Table 1). The median age of donors was 53 years (range 40–61). Donors had a median duration of infection of 18.6 years (range 6.0–24.4) and a median duration of suppressed viremia (<50 cp/mL) of 7.7 years (range 1.4–17.4). The median nadir CD4 was 293 cells/mm^3^ (range 18–576) and the median current CD4 was 729 cells/mm^3^ (range 461–1373). The median residual plasma viremia, measured by the single-copy assay [29], was 1.6 cp/mL (range <0.4–15.8). Some donors had insufficient cell yields from large volume phlebotomy (180mL whole blood) to assay all cell types simultaneously, so priority was given to PBMC, total and resting CD4^+^ T cells, in descending order. Flow cytometry staining was performed to assess the purity of the isolated cell subsets. Isolated total CD4^+^ T cells had a median 96% frequency of CD4^+^ T cells (range 93.2–98.3); resting CD4^+^ T cells had a median 96.9% frequency of CD4^+^ T cells (range 92.8–98.8) and a median 0.0% frequency of HLA-DR^+^ T cells (0.0–0.2).

**Fig 1:**
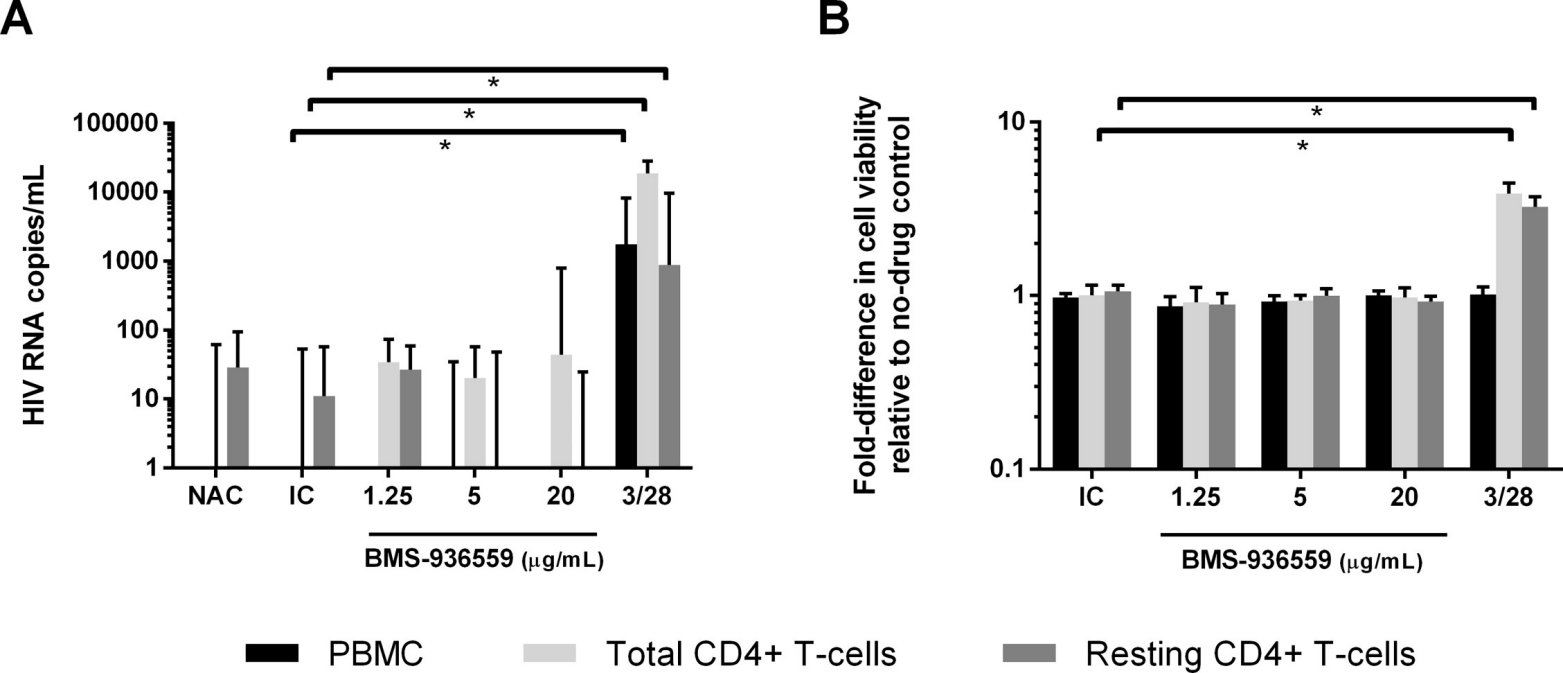
Virion production and cell viability in response to BMS-936559. Median HIV-1 virion production across all donors after 7 days of stimulation with BMS-936559 (Fig 1A). Median fold-change in cell viability across all donors from no-Ab control after 7 day treatment with BMS-936559 (Fig 1B). * = multiplicity adjusted, p<0.05 by Wilcoxon matched-pairs signed rank test with Bonferroni correction. NAC = no-Ab control; IC = isotype control; 3/28 = anti-CD3/CD28. Error bars = Interquartlie range (IQR).

For statistical analyses, virion production below the assay limit of detection (30 cp/mL culture supernatant) was considered non-detectable and set at 0 cp/mL. Cell viability was normalized to the no-Ab control for each cell population within each donor. Donor D1 had high levels of spontaneous virion production in the no-Ab and isotype controls (up to 15,290 cp/mL) and was excluded from analyses. The raw data for virion production is tabulated in S1 Table. Median HIV-1 RNA levels in supernatants from medium control cells for the other 11 donors was 50 cp/mL. Anti-CD3/CD28 treatment led to statistically significant increases in virion production from each cell population compared to isotype controls (PBMC: median 1746 cp/mL, p<0.01; total CD4^+^ T cells: median 18,773 cp/mL, p<0.01; resting CD4^+^ T cells: median 886 cp/mL, p<0.01; by Wilcoxon matched-pairs signed rank test) (Fig 1A, S1 Fig). Anti-CD3/CD28 stimulation also increased cell viability for total CD4^+^ and resting CD4^+^ T cell types (total CD4^+^ T cells: median 3.9-fold increase, p = 0.02; resting CD4^+^ T cells: median 3.3-fold increase, p = 0.03; by Wilcoxon matched-pairs signed rank test) (Fig 1B). No significant increases in cell viability of PBMC were observed following anti-CD3/CD28 stimulation. Across all donors, none of the concentrations of BMS-936559 resulted in statistically significant differences in virion production or cell viability compared to the isotype control for each cell population (Fig 1).

Despite the lack of a statistically significant overall virologic response to BMS-936559, we investigated whether cell types or certain BMS-936559 concentrations were associated with increased virus production (i.e., virus activation), defined as 2-fold greater HIV-1 RNA (cp/mL) in supernatants from cells treated with BMS-936559 compared to isotype control. If the isotype control led to virion production below the assay limit of detection (30 cp/mL of culture supernatant), then a response was defined as twice the limit of detection (>60 cp/mL). So defined, virus activation responses were observed for at least one concentration of BMS-936559 in at least one cell population from 7 of 11 donors. More specifically, virus activation responses were observed in PBMC from 4 of 11 donors (median 706 cp/mL for conditions producing virus activation), in total CD4^+^ T cells from 4 of 10 donors (median 81 cp/mL), and in resting CD4^+^ T cells from 1 of 8 donors (74 cp/mL). If responses were defined using a more stringent criteria of 3-fold increase in supernatant HIV-1 RNA compared to isotype control and >90 cp/mL, then virus activation was observed in PBMC from 4 of 11 donors (median 706 cp/mL), in total CD4^+^ T cells from 2 of 10 donors (median 7004 cp/mL), and in resting CD4^+^ T cells from 0 of 8 donors. Across all donors, the frequency of virus activation was not significantly different among the different cell types or across different BMS-936559 concentrations (p>0.05 by chi-squared test). HIV DNA frequency did not correlate with virus reactivation (p>0.05 by Mann-Whitney test).

To determine if virus activation responses to BMS-936559 were longitudinally reproducible, five donors (B3, W7, E5, C5, and K5) who showed >2-fold virus activation in one or more cell types had subsequent experiments performed with new blood draws using at least one of the cell populations that initially had responded to BMS-936559. The intervals between phlebotomies ranged from 3–26 months (median 15 months). Of the five donors with repeat experiments performed, only K5 had a reproducible response (S1 Fig). The first experiment with K5 showed 2,353 cp/mL of HIV-1 RNA in supernatants from total CD4^+^ T cells incubated with 20μg/mL BMS-936559. The second experiment with cells from this donor showed 683 cp/mL of HIV-1 RNA in supernatants from total CD4^+^ T cells incubated with 20 ug/mL of BMS-936559, as well as additional responses with 1.25 and 5 μg/mL BMS-936559 (391 and 62 HIV-1 RNA cp/mL, respectively). A third experiment with total CD4^+^ T cells from this donor did not show virus activation above isotype control at any BMS-936559 concentration. The proportions of PD-1 and PD-L1 expression varied by 0.6 to 7.2-fold amongst CD4^+^ and CD8^+^ T-cells across time points. These findings indicate that virus activation responses from BMS-936559 are infrequent, variable, and generally not reproducible in longitudinal samples.

Although virologic responses to BMS-936559 were not significant or reproducible, we hypothesized that blockade of PD-L1 alone may be insufficient to activate virus production and would require stimulation once the inhibitory effect of PD-L1 was removed. The PD-1 axis is known to suppress T cell receptor (TCR) signaling, so PD-1 axis blockade may increase HIV-1 latency reversal in T cells that are stimulated via the TCR [30,31]. To investigate this possibility, additional experiments were performed using cells from three donors who showed *>2*-fold virus activation responses to BMS-936559 (Donors W7, C5, K5). For each donor, the same cell populations that responded to BMS-936559 previously without additional stimulation were studied. Cells were stimulated using anti-CD3/CD28 antibody-coated beads (1 bead per cell) and cultured alone, with BMS-936559 at varying concentrations (1.25, 5, and 20 μg/mL), or with an isotype control. Virion production and cell viability were measured following seven days of culture, as described above, and were normalized relative to stimulation with anti-CD3/CD28 alone. None of the concentrations of BMS-936559 in anti-CD3/CD28-stimulated cells resulted in statistically significant increases in virion production or cell viability compared to isotype control (S3 Table).

### Blockade with anti-PD-1 (nivolumab)

The ability of PD-1 blockade using nivolumab, a fully human IgG_4_ anti-PD-1 mAb [23,32–40], to activate virus production was also evaluated *ex vivo*. The experimental methods were similar to above, except with modification of the cell types tested. Of the different cell populations assessed for virus activation responses to BMS-936559, PBMC and total CD4^+^ T cells had the greatest proportion of responses across donors. The greater response rates in PBMC may be caused by PD-L1 expression on antigen-presenting cells [41] and the greater response rates in total CD4^+^ T cells may be caused by the presence of activated CD4^+^ T cells [42,43]. Since blockade of the PD-1 axis can improve HIV-1-specific CD8^+^ T cell function [10], CD8-depleted PBMC were also evaluated for virus activation in response to nivolumab. The limited cell yields from large volume phlebotomies (180mL) did not permit assessment of all four cell populations, so resting CD4^+^ T cells were excluded from experiments with nivolumab because they had the fewest virus activation responses to BMS-936559.

Virus activation by nivolumab was assessed by culturing cells with either media alone, with nivolumab at varying concentrations that reflect therapeutic plasma concentrations (5 or 20 μg/mL) [32,40], or with isotype control, with or without anti-CD3/CD28 antibody-coated beads. Following seven days of treatment, virion production and cell viability were measured, as described above. The raw data for virion production is included in S3 Table. *Ex vivo* responses to nivolumab were evaluated in ten donors. Of these donors, the median age was 54.5 years (range 46–61). The median estimated duration of HIV-1 infection was 18.3 years (range 5.7–25.9) and the median duration of suppressed viremia (<50 cp/mL) was 8.2 years (range 4.5–15.8). Median nadir CD4 was 242 cells/mm^3^ (range 13–512) and the median current CD4 was 955 cells/mm^3^ (range 507–1426). The median residual plasma viremia was 1.1 cp/mL (range <0.4 to 10.7) (Table 1).

**Fig 2:**
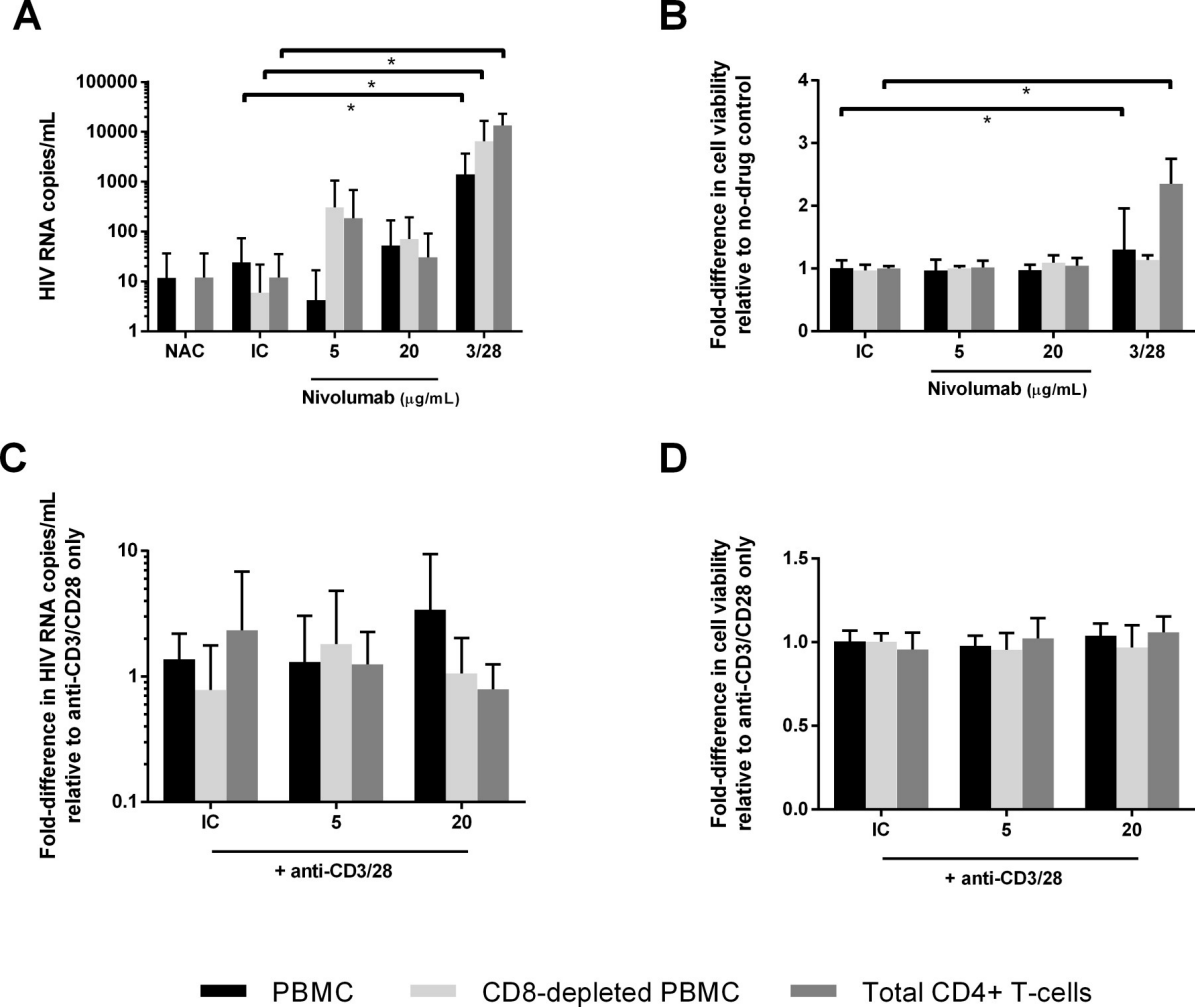
Virion production and cell viability in response to nivolumab. Median virion production across all donors in response to nivolumab (Fig 2A). Median-fold difference in cell viability across all donors in response to nivolumab or anti-CD3/CD28 stimulation compared to isotype control (Fig 2B). Median virion production in response to nivolumab in cells stimulated with anti-CD3/CD28 (Fig 2C). Median cell viability in response to nivolumab in cells stimulated with anti-CD3/CD28 (Fig 2D). NAC = no-Ab control; IC = isotype control; 3/28 = anti-CD3/CD28; nivo = nivolumab. Error bars = IQR.

Anti-CD3/CD28 stimulation alone led to significant increases in virion production relative to isotype control alone (0–11 cp/mL) for each cell population (Fig 2A, PBMC: median supernatant HIV-1 RNA 728 cp/mL, p<0.01; CD8-depleted PBMC: median 2,907 cp/mL, p = 0.02; total CD4^+^ T cells: median 10,233 cp/mL, p<0.01; by Wilcoxon matched-pairs signed rank test) and increased cell viability compared to isotype control in PBMC and total CD4^+^ T cells (PBMC: median 1.3-fold increase, p<0.01; total CD4^+^ T cells: median 2.5-fold increase, p<0.01; by Wilcoxon matched-pairs signed rank test). By contrast, incubation with nivolumab did not significantly increase virion production (Fig 2A) or cell viability (Fig 2B) across donors compared to isotype control for each of the cell populations tested (p>0.05 by Wilcoxon matched-pairs signed rank test).

In additional analyses, virus activation responses were defined as a 2-fold or greater increase in virion production from cells treated with nivolumab relative to cells treated with isotype control. As above, if the isotype control had virion production below the assay limit of detection (30 cp/mL) then a response was defined as twice the limit of detection (>60 cp/mL). For cells treated with nivolumab alone, 2-fold virus activation responses were observed for at least one concentration of nivolumab in at least one cell population from 3 of 9 donors. Responses were observed in PBMC from 2 of 9 donors (median 234 cp/mL in conditions with virologic responses), in CD8-depleted PBMC from 3 of 7 donors (median 248 cp/mL), and in total CD4^+^ T cells from 2 of 9 donors (median 185 cp/mL). When virologic responses were defined using stricter criteria of 3-fold increased virion production compared to isotype control and >90 cp/mL, virus activation was observed in PBMC from 1 of 9 donors (332 cp/mL), in CD8-depleted PBMC from 3 of 7 donors (median 299 cp/mL), and in total CD4^+^ T cells from 2 of 9 donors (median 185 cp/mL). Across all donors, no significant differences in virus activation responses were found between cell populations or among the different nivolumab concentrations (p>0.05 by Fisher’s exact test). HIV DNA level did not correlate with reactivation responses (p>0.05 by Mann-Whitney test). No difference in the frequencies of virus activation responses were observed between incubation with BMS-936559 alone and nivolumab alone (p>0.05 by Fisher’s exact test).

For analysis of experiments in which cells were treated with a combination of anti-CD3/CD28 and nivolumab, the virion production and cell viability levels were normalized relative to the anti-CD3/CD28 only treatment condition. No significant differences in virion production or viability were found in response to nivolumab as compared to isotype control in the anti-CD3/CD28-stimulated cells (p>0.05 by Wilcoxon matched-pairs signed rank test) (Fig 2C and Fig 2D).

### Immunophenotyping

The immunophenotype of PBMC was characterized using flow cytometry to measure the frequency of PD-1 and PD-L1 surface expression on CD3^+^CD4^+^ cells and CD3^+^CD4^neg^ (CD8^+^) cells (S4 Table). PD-1 and PD-L1 expression levels were detectable on CD4^+^ T cells and CD4^neg^ (CD8^+^) T cells in all donors. For the BMS-936559 experiments, the median PD-1 expression on CD8^+^ T-cells was 29.9% (range 19.3–42.5), median PD-1 expression on CD4^+^ T-cells was 46.1% (range 26.0–66.4), median PD-L1 expression on CD8^+^ T-cells was 26.71% (7.2–49.7), median PD-L1 expression on CD4^+^ T-cells was 31.1% (16.9–60.6). For the nivolumab experiments, the median PD-1 expression on CD8^+^ T-cells was 24.7% (range 11.7–30.1), median PD-1 expression on CD4^+^ T-cells was 35.2% (range 13.7–54.0), median PD-L1 expression on CD8^+^ T-cells was 5.3% (1.6–24.0), median PD-L1 expression on CD4^+^ T-cells was 15.9% (2.6–42.7). These values are consistent with previously reported values for HIV-infected individuals on ART [13,41,44–49].

Flow cytometry studies were completed in 3 batches along with health donor cells in each batch. The PD-1 expression range on CD8^+^ T-cells range was 32.4–44.9%, PD-1 expression range on CD4^+^ T-cells was 25.5–40.3%, PD-L1 expression range on CD8^+^ T-cells was 3.6–30.4%, PD-L1 expression range on CD4^+^ T-cells was 11.4–35.6%.

For nivolumab, virus reactivation responses, by either the 2-fold or 3-fold increase definitions, correlated with baseline PD-1 expression on CD4^+^ T-cells (P = 0.047), PD-L1 expression on CD8^+^ T-cells (P = 0.024), and PD-L1 expression on CD4^+^ T-cells (P = 0.047) by the Mann-Whitney test. For BMS-936559, virus reactivation responses, by the 3-fold increase in virion production definition, correlated with PD-1 expression on CD4^+^ T-cells (P = 0.0303) by Mann-Whitney test. These correlations became insignificant after the Bonferroni correction.

## Discussion

Immune checkpoint blockade can promote anti-HIV-1 immunity [10,16–19] but may also possess the desired property of proviral activation (i.e., latency reversal). CD4^+^ T cells expressing immune checkpoint markers (PD-1, TIGIT, LAG3) are enriched for HIV-1-infected cells and correlate with the number of HIV-1 infected cells in individuals receiving ART [13,44,45,50–52]. Therefore, immune checkpoint blockade could impact the HIV-1 reservoir by destabilizing proviral latency and enhancing proviral expression. This concept was demonstrated in viremic and aviremic SIV-infected rhesus macaques wherein *in vivo* checkpoint blockade with anti-PD-1 antibody induced transient increases in SIV viremia [53,54]. Observed responses were associated with CD4^+^ T cell proliferation [53], suggesting that PD-1 blockade promoted T cell activation that led to latency reversal. By contrast, other *in vivo* macaque studies showed no change in viremia when disrupting the PD-1 axis [55,56].

Preliminary *ex vivo* human studies have reported that PD-1 blockade can increase virion production in CD4^+^ T cells from HIV-1-infected viremic donors [57] and in CD4^+^ T cells with allogeneic cell or cytokine stimulation (IL-7, IL-15) from donors on suppressive ART [58]. A number of case studies of HIV-1-infected individuals with NSCLC or malignant melanoma who were treated with nivolumab or ipilimumab (anti-CTLA4) reported changes in HIV-1 RNA or DNA [17–19,59]. By contrast, other studies investigating immune checkpoint blockade for the treatment of cancer in HIV-1-infected individuals have not observed changes in plasma HIV-1 RNA and only inconsistent changes in HIV-1 DNA [17–19,24,26,59]. No changes in residual viremia or cell-associated HIV-1 RNA were observed in HIV-1-infected participants on effective ART without concomitant malignancy in a phase I, randomized, placebo-controlled, single-dose study of BMS-936559 [16]. Results from this clinical trial did not reveal any changes in residual viremia or cell-associated HIV-1 DNA or RNA in 6 of 6 patients treated with 0.3 mg/kg BMS-936559, although there was evidence of improved HIV-1-specific CD8^+^ T cell function in 2 of 6 participants [16]. The dose in this study was lower than the lowest evaluated doses in phase I clinical studies for cancer [27] and may have been too low to increase virus production.

In the current *ex vivo* study, BMS-936559 and nivolumab were evaluated at concentrations that reflect therapeutic plasma concentrations and are associated with PD-L1 and PD-1 receptor occupancy of ≥70%, respectively [27,32,40,60]. We conducted analyses in which virus activation was defined as 2-fold increases in HIV-1 RNA compared to cells treated with isotype control. So defined, virus activation responses in at least one cell type were observed in 7 of 11 donors incubated with BMS-936559 and in 3 of 9 donors with cells treated with nivolumab. However, responses were inconsistent, occurring in varying cell types and at different antibody concentrations. Responses also did not correlate with HIV DNA levels. Baseline PD-1 and PD-L1 expression correlated with virus production, although these results should be re-evaluated in future studies given the small sample size of this study.

Recent studies have shown that the PD-1 axis suppresses CD28 signaling [30] and that effective PD-1 targeted therapy for restoration of CD8^+^ T cell function requires CD28 co-stimulation [31]. Yet, in our study *ex vivo* virologic responses to PD-1/L1 blockade were not increased by TCR/CD28 stimulation. It is possible that the stimulation used in this study via anti-CD3/28 coated beads was too potent to observe an augmented response with PD-1/L1 blockade as compared to prior studies, that used allogeneic cell or cytokine stimulation (IL-7, IL-15) [57,58].

The absence of a consistent effect of PD-1 axis blockade on virus production among the donors studied is likely multifactorial. First, although PD-1 expression is enriched in HIV-1 infected cells, many HIV-1 infected cells do not express PD-1 and would therefore not be expected to respond to PD-1 axis blockade [13,44]. Second, HIV-1 infected cells that express PD-1 may be resistant to PD-1 axis blockade from severe exhaustion [61] and upregulation of additional immune checkpoints [13,61]. Co-expression of multiple immune checkpoint molecules is associated with higher frequencies of HIV-infected cells compared to expression of single immune checkpoints [13], consequently, inhibition of multiple immune checkpoints may be required for more effective HIV-1 latency reversal. Combination blockade of multiple inhibitory receptors has been shown to improve immune responses, although the associated toxicities will limit this approach [23,62,63]. Third, it is possible that testing more cells or using more sensitive assays of proviral activation, such as measuring HIV-1 RNA transcripts, could have revealed more subtle effects of immune checkpoint blockade than by measuring virion production. Fourth, the infrequent and variable responses that were not longitudinally reproduced in this study suggests that responses are stochastic, possibly from spontaneous viral activation. It is possible that concomitant stimulation may be required to observe the effects of PD-1 blockade, as observed in previous studies [57,58]. Finally, *in vivo* studies, including mouse models, may allow for more rigorous testing of immune checkpoint blockade. Further human clinical trials will be the best determinant of whether immune checkpoint blockade is safe and can meaningfully impact the HIV-1 reservoir, and whether these therapies are safe for people living with HIV-1.

## Methods

### Quantification of residual plasma viremia by single-copy assay

Plasma was harvested by centrifugation of whole blood at 400 x g for 10 min, followed by additional centrifugation of plasma at 1350 x g for 15 min. Cell-free plasma was stored at -80°C. HIV-1 RNA was quantified using a two-step qRT-PCR assay that targets the integrase region of *pol* [29]. The assay limit of detection is 1 copy of RNA per qPCR reaction.

### Isolation of PBMC, CD8-depleted PBMC, total CD4^+^ T cells, and resting CD4^+^ T cells

Large-volume phlebotomies (180 mL) were performed on patients who were on suppressive ART for ≥16 months. PBMC were isolated by Ficoll-Paque density gradient centrifugation. CD8^+^ T cells were removed from PBMC by positive selection using BD iMag anti-human CD8 Particles (BD). Total CD4^+^ T cells were isolated from PBMC by negative selection using the EasySepCD4^+^ T cell Enrichment Kit (STEMCELL). Resting CD4^+^ T cells were isolated from PBMC by negative selection using the EasySep Human Custom Enrichment Kit (STEMCELL) that depletes cells expressing CD8, CD14, CD16, CD19, CD20, CD36, CD56, CD66b, CD123, TCRγ/δ, glycophorin A, CD69, CD25, and HLA-DR.

### Cell culture

PBMC, CD8-depleted PBMC, total CD4^+^ T cells, and/or resting CD4^+^ T cells were seeded into 48-well plates at 1x10^6^ cells/mL with up to three replicates. Cells were cultured for 7 days with RPMI 1640 medium supplemented with 10% (vol/vol) FBS, 2mM L-Glutamine, 0.6% penicillin/streptomycin, and 300nM each of efavirenz and raltegravir to prevent new rounds of infection. Cultures were performed for 7 days because this was the peak of virion accumulation in preliminary studies with anti-CD3/CD28 stimulation. Cells were treated with varying concentrations of BMS-936559 (1.25, 5, or 20μg/mL), varying concentrations of nivolumab (5 or 20μg/mL), or IgG_4_ isotype control (20μg/mL) with or without concurrent incubation with anti-CD3/CD28 antibody-coated beads at a concentration of 1 bead/cell (Dynabeads Human T-Activator CD3/CD28, Invitrogen).

### Quantification of virion HIV-1 RNA in cell culture supernatant

After 7 days of culture, supernatants from replicate wells were collected and pooled. HIV-1 RNA was quantified using the Roche Cobas AmpliPrep/Roche Cobas Taqman v2.0 (CAP/CTM). The assay limit of detection is 20 cp per reaction, which scaled to 30 cp/mL of culture supernatant. Supernatants from cultures of fresh cells measured using CAP/CTM have previously been shown to be free of contaminating HIV-1 DNA [28,64].

### Quantification of HIV DNA by qPCR

Cells were stored in 1x10^6^ aliquots at -80°C. HIV DNA was quantified as previously described [65].

### Assays of cellular viability

Day 7 cells were assessed for viability using CellTiter Fluor (Promega). Fluorescence was read at excitation/emission 390/505nm.

### Flow cytometric analyses

Cellular purities of isolated total CD4^+^ T cells, resting CD4^+^ T cells, and CD8-depleted PBMC were assessed by flow cytometry. The purity of total CD4^+^ T cells and resting CD4^+^ T cells were compared to PBMC by staining aliquots of 1x10^6^ PBMC, purified total CD4^+^ T cells, and resting CD4^+^ T cells with FITC anti-CD4 (RPA-T4, BD), FITC isotype control (mouse IgG_1κ_, BD), PerCP-Cy5.5 anti-HLA-DR (G46-6, BD), or PerCP-Cy5.5 isotype control (mouse IgG_2aκ_, BD) antibodies. CD8 depletion was assessed in aliquots of 1x10^6^ PBMC and CD8-depleted PBMC stained with V450 CD3 (UCHT1, BD) and PerCP-Cy5.5 CD8 (RPA-T8, BD). Data were acquired using an LSRII cytometer and FACSDiva software (BD). At least 20,000 events were collected from all samples. Fluorescence minus one and isotypes controls were used to set gates for analysis. The flow cytometry gating strategy is summarized in S2 Fig.

Baseline expression of PD-1 and PD-L1 on PBMC was determined on aliquots of 0.5-1x10^6^ PBMC isolated on the day of phlebotomy. Samples were incubated with anti-PD-L1 primary antibody (BMS-936559, Bristol-Myers Squibb) or isotype control (human IgG_4_, Bristol-Myers Squibb) then with V500 CD3 (UCHT1, BD), PerCP CD4 (L200, BD), BV421 anti-PD-1 (EH12.2H7, BioLegend), BV421 isotype control (mouse IgG_1κ_ BioLegend), and anti-PD-L1 secondary antibodies (mouse anti-hIgG_4_ PE, abcam). Following staining, samples were washed with stain buffer twice and cells were fixed using BD Cytofix buffer. Live singlet cells were identified by live-dead staining and forward scatter profiles. Fluorescence minus one controls were used to set gates for analysis.

### Ethics statement

All study participants provided written, informed consent. All original, signed consent forms are maintained in the study file and all procedures were approved by the University of Pittsburgh Institutional Review Board.

## Supporting information

S1 Fig Longitudinal responses to BMS-936559. Donors B3 (Panel A), W7 (Panel B), E5 (Panel C), C5 (Panel D), and K5 (Panel E) had repeat experiments using at least one of the cell populations that initially showed latency reversal responses to BMS-936559. Of the five donors with longitudinal experiments performed, only donor K5 had a reproducible virus activation response to BMS-936559 for total CD4^+^ T cells in the experiment performed with the second blood draw but not the third blood draw (Panel E). HIV RNA (copies/mL) shown are in culture supernatants of the cells and incubation conditions indicated. Total CD4 = total CD4^+^ T cells; Resting CD4 = resting CD4^+^ T cells; PBMC = Peripheral blood mononuclear cells; NAC = no-Ab control; IC = isotype control; * = not done.
(DOCX)

S2 Fig Flow cytometry gating strategy. This schematic illustrates how CD4+ and CD8+ (CD4-) T-cells were gated by flow cytometry to measure PD-1 and PD-L1 expression.
(DOCX)

S1 Table Virion production in response to BMS-936559. Virion production as HIV RNA copies/mL. Cells with yellow shading have virologic responses when defined as being greater than twice the virion production from cells treated with isotype control or > 60 copies/mL. Cells with bolded font have virologic responses when defined as being greater than three times the virion production from cells treated with isotype control or = > 90 copies/mL. BMS = BMS-936559, IC = isotype control, AC = activation control with anti-CD3/28, TND = HIV-1 RNA target not detected.
(DOCX)

S2 Table Virion production in cells stimulated with anti-CD3/CD28 antibodies and BMS-936559. Virion production as HIV RNA copies/mL. 3/28 = anti-CD3/28, IC = isotype control, BMS = BMS-936559, TND = target not detected.
(DOCX)

S3 Table Virion production in response to nivolumab. Virion production as HIV RNA copies/mL. Cells with yellow background have virologic responses when defined as being greater than twice the virion production from cells treated with isotype control or as > 60 copies/mL. Cells with bolded font have virologic responses when defined as being greater than three times the virion production from cells treated with isotype control or as > 90 copies/mL. IC = isotype control, nivo = nivolumab, AC = activation control with anti-CD3/28, TND = target not detected.
(DOCX)

S4 Table PD-1 and PD-L1 expression by flow cytometry. The proportion of cells expressing PD-1 or PD-L1 were measured by flow cytometry on CD4+ T-cells and CD8+ T-cells. N/A = Not Applicable.
(DOCX)

## References

[1] Finzi D, Hermankova M, Pierson T, Carruth LM, Buck C, Chaisson RE, et al Identification of a reservoir for HIV-1 in patients on highly active antiretroviral therapy. Science. 1997;278: 1295–1300. 9360927 10.1126/science.278.5341.1295

[2] Wong JK, Hezareh M, Gunthard HF, Havlir DV, Ignacio CC, Spina CA, et al Recovery of replication-competent HIV despite prolonged suppression of plasma viremia. Science. 1997;278: 1291–1295. 9360926 10.1126/science.278.5341.1291

[3] Finzi D, Blankson J, Siliciano JD, Margolick JB, Chadwick K, Pierson T, et al Latent infection of CD4+ T cells provides a mechanism for lifelong persistence of HIV-1, even in patients on effective combination therapy. Nat Med. 1999;5: 512–517. 10.1038/8394 10229227

[4] Chun TW, Carruth L, Finzi D, Shen X, DiGiuseppe JA, Taylor H, et al Quantification of latent tissue reservoirs and total body viral load in HIV-1 infection. Nature. 1997;387: 183–188. 10.1038/387183a0 9144289

[5] Siliciano JD, Kajdas J, Finzi D, Quinn TC, Chadwick K, Margolick JB, et al Long-term follow-up studies confirm the stability of the latent reservoir for HIV-1 in resting CD4+ T cells. Nat Med. 2003;9: 727–728. 10.1038/nm880 12754504

[6] Kearney MF, Wiegand A, Shao W, Coffin JM, Mellors JW, Lederman M, et al Origin of Rebound Plasma HIV Includes Cells with Identical Proviruses That Are Transcriptionally Active before Stopping of Antiretroviral Therapy. J Virol. 2015;90: 1369–1376. 10.1128/JVI.02139-15 26581989 PMC4719635

[7] Joos B, Fischer M, Kuster H, Pillai SK, Wong JK, Boni J, et al HIV rebounds from latently infected cells, rather than from continuing low-level replication. Proc Natl Acad Sci U S A. 2008;105: 16725–16730. 10.1073/pnas.0804192105 18936487 PMC2575487

[8] Kearney MF, Spindler J, Shao W, Yu S, Anderson EM, O'Shea A, et al Lack of detectable HIV-1 molecular evolution during suppressive antiretroviral therapy. PLoS Pathog. 2014;10: e1004010 10.1371/journal.ppat.1004010 24651464 PMC3961343

[9] Deeks SG, Lewin SR, Ross AL, Ananworanich J, Benkirane M, Cannon P, et al International AIDS Society global scientific strategy: towards an HIV cure 2016. Nat Med. 2016;22: 839–850. 10.1038/nm.4108 27400264 PMC5322797

[10] Bui JK, Mellors JW. Reversal of T-cell exhaustion as a strategy to improve immune control of HIV-1. AIDS. 2015;29: 1911–1915. 10.1097/QAD.0000000000000788 26355569

[11] Pardoll DM. The blockade of immune checkpoints in cancer immunotherapy. Nat Rev Cancer. 2012;12: 252–264. 10.1038/nrc3239 22437870 PMC4856023

[12] Chew GM, Fujita T, Webb GM, Burwitz BJ, Wu HL, Reed JS, et al TIGIT Marks Exhausted T Cells, Correlates with Disease Progression, and Serves as a Target for Immune Restoration in HIV and SIV Infection. PLoS Pathog. 2016;12: e1005349 10.1371/journal.ppat.1005349 26741490 PMC4704737

[13] Fromentin R, Bakeman W, Lawani MB, Khoury G, Hartogensis W, DaFonseca S, et al CD4+ T Cells Expressing PD-1, TIGIT and LAG-3 Contribute to HIV Persistence during ART. PLoS Pathog. 2016;12: e1005761 10.1371/journal.ppat.1005761 27415008 PMC4944956

[14] Hoffmann M, Pantazis N, Martin GE, Hickling S, Hurst J, Meyerowitz J, et al Exhaustion of Activated CD8 T Cells Predicts Disease Progression in Primary HIV-1 Infection. PLoS Pathog. 2016;12: e1005661 10.1371/journal.ppat.1005661 27415828 PMC4945085

[15] Hurst J, Hoffmann M, Pace M, Williams JP, Thornhill J, Hamlyn E, et al Immunological biomarkers predict HIV-1 viral rebound after treatment interruption. Nat Commun. 2015;6: 8495 10.1038/ncomms9495 26449164 PMC4633715

[16] Gay CL, Bosch RJ, Ritz J, Hataye JM, Aga E, Tressler RL, et al Clinical Trial of the Anti-PD-L1 Antibody BMS-936559 in HIV-1 Infected Participants on Suppressive Antiretroviral Therapy. J Infect Dis. 2017;215: 1725–1733. 10.1093/infdis/jix191 28431010 PMC5790148

[17] Guihot A, Marcelin AG, Massiani MA, Samri A, Soulie C, Autran B, et al Drastic decrease of the HIV reservoir in a patient treated with nivolumab for lung cancer. Ann Oncol. 2018;29: 517–518. 10.1093/annonc/mdx696 29206889

[18] Le Garff G, Samri A, Lambert-Niclot S, Even S, Lavole A, Candranel J, et al Transient HIV-specific T cells increase and inflammation in an HIV-infected patient treated with nivolumab. AIDS. 2017;31: 1048–1051. 10.1097/QAD.0000000000001429 28350581

[19] Samri A, Lavolé A, Even S, Lambert-Niclot S, Le Garff G, Candranel J, et al Immunovirological evolution in HIV-infected patients treated with anti-PD1 therapy International AIDS Society (IAS) 2017 Paris, France. Abstract Number: MOPEB0362.

[20] Sharma P, Allison JP. The future of immune checkpoint therapy. Science. 2015;348: 56–61. 10.1126/science.aaa8172 25838373

[21] Callahan MK, Postow MA, Wolchok JD. Targeting T Cell Co-receptors for Cancer Therapy. Immunity. 2016;44: 1069–1078. 10.1016/j.immuni.2016.04.023 27192570

[22] Robert C, Schachter J, Long GV, Arance A, Grob JJ, Mortier L, et al Pembrolizumab versus Ipilimumab in Advanced Melanoma. N Engl J Med. 2015;372: 2521–2532. 10.1056/NEJMoa1503093 25891173

[23] Larkin J, Chiarion-Sileni V, Gonzalez R, Grob JJ, Cowey CL, Lao CD, et al Combined Nivolumab and Ipilimumab or Monotherapy in Untreated Melanoma. N Engl J Med. 2015;373: 23–34. 10.1056/NEJMoa1504030 26027431 PMC5698905

[24] Heppt MV, Schlaak M, Eigentler TK, Kahler KC, Kiecker F, Loquai C, et al Checkpoint blockade for metastatic melanoma and Merkel cell carcinoma in HIV-positive patients. Ann Oncol. 2017;28: 3104–3106. 10.1093/annonc/mdx538 28950303

[25] Morris VK, Salem ME, Nimeiri H, Iqbal S, Singh P, Ciombor K. et al Nivolumab for previously treated unresectable metastatic anal cancer (NCI9673): a multicentre, single-arm, phase 2 study. Lancet Oncol. 2017;18: 446–453. 10.1016/S1470-2045(17)30104-3 28223062 PMC5809128

[26] Davar D, Wilson M, Pruckner C, Kirkwood JM. PD-1 Blockade in Advanced Melanoma in Patients with Hepatitis C and/or HIV. Case Rep Oncol Med. 2015;2015: 737389 10.1155/2015/737389 26448890 PMC4581502

[27] Brahmer JR, Tykodi SS, Chow LQ, Hwu WJ, Topalian SL, Hwu P, et al Safety and activity of anti-PD-L1 antibody in patients with advanced cancer. N Engl J Med. 2012;366: 2455–2465. 10.1056/NEJMoa1200694 22658128 PMC3563263

[28] Cillo AR, Hong F, Tsai A, Irrinki A, Kaur J, Sloan DD, et al Blood biomarkers of expressed and inducible HIV-1. AIDS. 2018;32: 699–708. 10.1097/QAD.0000000000001748 29334544 PMC5854535

[29] Cillo AR, Vagratian D, Bedison MA, Anderson EM, Kearney MF, Fyne E, et al Improved single-copy assays for quantification of persistent HIV-1 viremia in patients on suppressive antiretroviral therapy. J Clin Microbiol. 2014;52: 3944–3951. 10.1128/JCM.02060-14 25187636 PMC4313209

[30] Hui E, Cheung J, Zhu J, Su X, Taylor MJ, Wallweber HA, et al T cell costimulatory receptor CD28 is a primary target for PD-1-mediated inhibition. Science. 2017;355(6332): 1428–1433. 10.1126/science.aaf1292 28280247 PMC6286077

[31] Kamphorst AO, Wieland A, Nasti T, Yang S, Zhang R, Barber DL, et al Rescue of exhausted CD8 T cells by PD-1-targeted therapies is CD28-dependent. Science. 2017;355(6332):1423–1427. 10.1126/science.aaf0683 28280249 PMC5595217

[32] Brahmer JR, Drake CG, Wollner I, Powderly JD, Picus J, Sharfman WH, et al Phase I study of single-agent anti-programmed death-1 (MDX-1106) in refractory solid tumors: safety, clinical activity, pharmacodynamics, and immunologic correlates. J Clin Oncol. 2010;28: 3167–3175. 10.1200/JCO.2009.26.7609 20516446 PMC4834717

[33] Topalian SL, Hodi FS, Brahmer JR, Gettinger SN, Smith DC, McDermott DF, et al Safety, activity, and immune correlates of anti-PD-1 antibody in cancer. N Engl J Med. 2012;366: 2443–2454. 10.1056/NEJMoa1200690 22658127 PMC3544539

[34] Topalian SL, Sznol M, McDermott DF, Kluger HM, Carvajal RD, Sharfman WH, et al Survival, durable tumor remission, and long-term safety in patients with advanced melanoma receiving nivolumab. J Clin Oncol. 2014;32: 1020–1030. 10.1200/JCO.2013.53.0105 24590637 PMC4811023

[35] Robert C, Long GV, Brady B, Dutriaux C, Maio M, Mortier L, et al Nivolumab in previously untreated melanoma without BRAF mutation. N Engl J Med. 2015;372: 320–330. 10.1056/NEJMoa1412082 25399552

[36] Schadendorf D, Hodi FS, Robert C, Weber JS, Margolin K, Hamid O, et al Pooled Analysis of Long-Term Survival Data From Phase II and Phase III Trials of Ipilimumab in Unresectable or Metastatic Melanoma. J Clin Oncol. 2015;33: 1889–1894. 10.1200/JCO.2014.56.2736 25667295 PMC5089162

[37] Brahmer J, Reckamp KL, Baas P, Crino L, Eberhardt WE, Poddubskaya E, et al Nivolumab versus Docetaxel in Advanced Squamous-Cell Non-Small-Cell Lung Cancer. N Engl J Med. 2015;373: 123–135. 10.1056/NEJMoa1504627 26028407 PMC4681400

[38] Borghaei H, Paz-Ares L, Horn L, Spigel DR, Steins M, Ready NE, et al Nivolumab versus Docetaxel in Advanced Nonsquamous Non-Small-Cell Lung Cancer. N Engl J Med. 2015;373: 1627–1639. 10.1056/NEJMoa1507643 26412456 PMC5705936

[39] Ansell SM, Lesokhin AM, Borrello I, Halwani A, Scott EC, Gutierrez M, et al PD-1 blockade with nivolumab in relapsed or refractory Hodgkin's lymphoma. N Engl J Med. 2015;372: 311–319. 10.1056/NEJMoa1411087 25482239 PMC4348009

[40] Yamamoto N, Nokihara H, Yamada Y, Shibata T, Tamura Y, Seki Y, et al Phase I study of Nivolumab, an anti-PD-1 antibody, in patients with malignant solid tumors. Invest New Drugs. 2017;35: 207–216. 10.1007/s10637-016-0411-2 27928714 PMC5352798

[41] Sachdeva M, Fischl MA, Pahwa R, Sachdeva N, Pahwa S. Immune exhaustion occurs concomitantly with immune activation and decrease in regulatory T cells in viremic chronically HIV-1-infected patients. J Acquir Immune Defic Syndr. 2010;54: 447–454. 10.1097/QAI.0b013e3181e0c7d0 20463584 PMC3095513

[42] Tumeh PC, Harview CL, Yearley JH, Shintaku IP, Taylor EJ, Robert L, et al PD-1 blockade induces responses by inhibiting adaptive immune resistance. Nature. 2014;515: 568–571. 10.1038/nature13954 25428505 PMC4246418

[43] Rizvi NA, Hellmann MD, Snyder A, Kvistborg P, Makarov V, Havel JJ, et al Cancer immunology. Mutational landscape determines sensitivity to PD-1 blockade in non-small cell lung cancer. Science. 2015; 348: 124–128. 10.1126/science.aaa1348 25765070 PMC4993154

[44] Chomont N, El-Far M, Ancuta P, Trautmann L, Procopio FA, Yassine-Diab B, et al HIV reservoir size and persistence are driven by T cell survival and homeostatic proliferation. Nat Med. 2009;15: 893–900. 10.1038/nm.1972 19543283 PMC2859814

[45] Hatano H, Jain V, Hunt PW, Lee TH, Sinclair E, Do TD, et al Cell-based measures of viral persistence are associated with immune activation and programmed cell death protein 1 (PD-1)-expressing CD4+ T cells. J Infect Dis. 2013;208: 50–56. 10.1093/infdis/jis630 23089590 PMC3666131

[46] Zhang JY, Zhang Z, Wang X, Fu JL, Yao J, Jiao Y, et al PD-1 up-regulation is correlated with HIV-specific memory CD8+ T-cell exhaustion in typical progressors but not in long-term nonprogressors. Blood. 2007;109: 4671–4678. 10.1182/blood-2006-09-044826 17272504

[47] Nakayama K, Nakamura H, Koga M, Koibuchi T, Fujii T, Miura T, et al Imbalanced production of cytokines by T cells associates with the activation/exhaustion status of memory T cells in chronic HIV type 1 infection. AIDS Res Hum Retroviruses. 2012;28: 702–714. 10.1089/AID.2011.0073 21902582

[48] Day CL, Kaufmann DE, Kiepiela P, Brown JA, Moodley ES, Reddy S, et al PD-1 expression on HIV-specific T cells is associated with T-cell exhaustion and disease progression. Nature. 2006;443: 350–354. 10.1038/nature05115 16921384

[49] Yamamoto T, Price DA, Casazza JP, Ferrari G, Nason M, Chattopadhyay PK, et al Surface expression patterns of negative regulatory molecules identify determinants of virus-specific CD8+ T-cell exhaustion in HIV infection. Blood. 2011;117: 4805–4815. 10.1182/blood-2010-11-317297 21398582 PMC3100691

[50] Pallikkuth S, Sharkey M, Babic DZ, Gupta S, Stone GW, Fischl MA, et al Peripheral T Follicular Helper Cells Are the Major HIV Reservoir within Central Memory CD4 T Cells in Peripheral Blood from Chronically HIV-Infected Individuals on Combination Antiretroviral Therapy. J Virol. 2015;90: 2718–2728. 10.1128/JVI.02883-15 26676775 PMC4810658

[51] Banga R, Procopio FA, Noto A, Pollakis G, Cavassini M, Ohmiti K, et al PD-1(+) and follicular helper T cells are responsible for persistent HIV-1 transcription in treated aviremic individuals. Nat Med. 2016;22: 754–761. 10.1038/nm.4113 27239760

[52] Perreau M, Savoye AL, De Crignis E, Corpataux JM, Cubas R, Haddad EK, et al Follicular helper T cells serve as the major CD4 T cell compartment for HIV-1 infection, replication, and production. J Exp Med. 2013;210: 143–156. 10.1084/jem.20121932 23254284 PMC3549706

[53] Velu V, Titanji K, Zhu B, Husain S, Pladevega A, Lai L, et al Enhancing SIV-specific immunity in vivo by PD-1 blockade. Nature. 2009;458: 206–210. 10.1038/nature07662 19078956 PMC2753387

[54] Finnefrock AC, Tang A, Li F, Freed DC, Feng M, Cox KS, et al PD-1 blockade in rhesus macaques: impact on chronic infection and prophylactic vaccination. J Immunol. 2009;182: 980–987. 19124741 10.4049/jimmunol.182.2.980

[55] Mason S, Tenney D, Balsitis S, Rose B, Levine S, Campellone S, et al. Dual approach to HIV-1 cure: Activation of latency and restoration of exhausted virus-specific T cell function. 6th HIV Persistence Workshop, Session IX: Drug Discovery. 2013. Miami, Florida, USA. Abstract Number: 44.

[56] Amancha PK, Hong JJ, Rogers K, Ansari AA, Villinger F. In vivo blockade of the programmed cell death-1 pathway using soluble recombinant PD-1-Fc enhances CD4+ and CD8+ T cell responses but has limited clinical benefit. J Immunol. 2013;191: 6060–6070. 10.4049/jimmunol.1302044 24227774 PMC3858463

[57] DaFonseca S, Chomont N, El Far M, Boulassel R, Routy J, Sekaly R. Purging the HIV-1 reservoir through the disruption of the PD-1 pathway. J Int AIDS Soc. 2010;13(Suppl 3): O15.

[58] Chomont N. Immunologic Strategies to Cure HIV Infection. International Workshop on HIV and Hepatitis Virus Drug Resistance and Curative Strategies. 2013. Toronto, Ontario, Canada. Plenary Abstract Number P2.

[59] Tomsitz D, Hein R, Biedermann T, Kohlmeyer J. Treatment of a patient with HIV and metastatic melanoma with consequitive ipilimumab and nivolumab. J Eur Acad Dermatol Venereol. 2017;32(1):e26–e28. 10.1111/jdv.14450 28662283

[60] Eron J, Gay C, Bosch R, Ritz J, Hataye J, Hwang C, et al. Safety, Immunologic and Virologic Activity of Anti-PD-L1 in HIV-1 Participants on ART. Session: Viral Reservoirs/Antiretroviral Therapy Randomized Clinical Trials. Conference on Retroviruses and Opportunistic Infections (CROI). 2016. Boston, Massachusetts, USA. Session Number: O-2.

[61] Wherry EJ, Kurachi M. Molecular and cellular insights into T cell exhaustion. Nat Rev Immunol. 2015;15: 486–499. 10.1038/nri3862 26205583 PMC4889009

[62] Postow MA, Chesney J, Pavlick AC, Robert C, Grossmann K, McDermott D, et al Nivolumab and ipilimumab versus ipilimumab in untreated melanoma. N Engl J Med. 2015;372: 2006–2017. 10.1056/NEJMoa1414428 25891304 PMC5744258

[63] Grabmeier-Pfistershammer K, Stecher C, Zettl M, Rosskopf S, Rieger A, Zlabinger GJ, et al Antibodies targeting BTLA or TIM-3 enhance HIV-1 specific T cell responses in combination with PD-1 blockade. Clin Immunol. 2017;183: 167–173. 10.1016/j.clim.2017.09.002 28882621

[64] Wei DG, Chiang V, Fyne E, Balakrishnan M, Barnes T, Graupe M, et al Histone deacetylase inhibitor romidepsin induces HIV expression in CD4 T cells from patients on suppressive antiretroviral therapy at concentrations achieved by clinical dosing. PLoS Pathog. 2014;10: e1004071 10.1371/journal.ppat.1004071 24722454 PMC3983056

[65] Hong F, Aga E, Cillo AR, Yates AL, Besson G, Fyne E, et al Novel Assays for Measurement of Total Cell-Associated HIV-1 DNA and RNA. J Clin Microbiol. 2016;54: 902–911. 10.1128/JCM.02904-15 26763968 PMC4809955

