# Supplementary material for: Blockade of the PD-1 axis alone is not sufficient to activate HIV-1 virion production from CD4+ T cells of individuals on suppressive ART
Source: PLoS One. 2019 Jan 25;14(1):e0211112. doi: 10.1371/journal.pone.0211112 (PMC6347234; doi:10.1371/journal.pone.0211112)
Supplement: S1 Fig — Donors B3 (Panel A), W7 (Panel B), E5 (Panel C), C5 (Panel D), and K5 (Panel E) had repeat experiments using at least one of the cell populations that initially showed latency reversal responses to BMS-936559. Of the five donors with longitudinal experiments performed, only donor K5 had a reproducible virus activation response to BMS-936559 for total CD4+ T cells in the experiment performed with the second blood draw but not the third blood draw (Panel E). HIV RNA (copies/mL) shown are in culture supernatants of the cells and incubation conditions indicated. Total CD4 = total CD4+ T cells; Resting CD4 = resting CD4+ T cells; PBMC = Peripheral blood mononuclear cells; NAC = no-Ab control; IC = isotype control; * = not done. (DOCX) [file pone.0211112.s001.docx]

**S1 Fig** **Longitudinal Responses to BMS-936559**

Donors B3 (Panel A), W7 (Panel B), E5 (Panel C), C5 (Panel D), and K5 (Panel E) had repeat experiments using at least one of the cell populations that initially showed latency reversal responses to BMS-936559. Of the five donors with longitudinal experiments performed, only donor K5 had a reproducible virus activation response to BMS-936559 for total CD4^+^ T cells in the experiment performed with the second blood draw but not the third blood draw (Panel E). HIV RNA (copies/mL) shown are in culture supernatants of the cells and incubation conditions indicated. Total CD4 = total CD4^+^ T cells; Resting CD4 = resting CD4^+^ T cells; PBMC = Peripheral blood mononuclear cells; NAC = no-Ab control; IC = isotype control; * = not done.

**A**

**
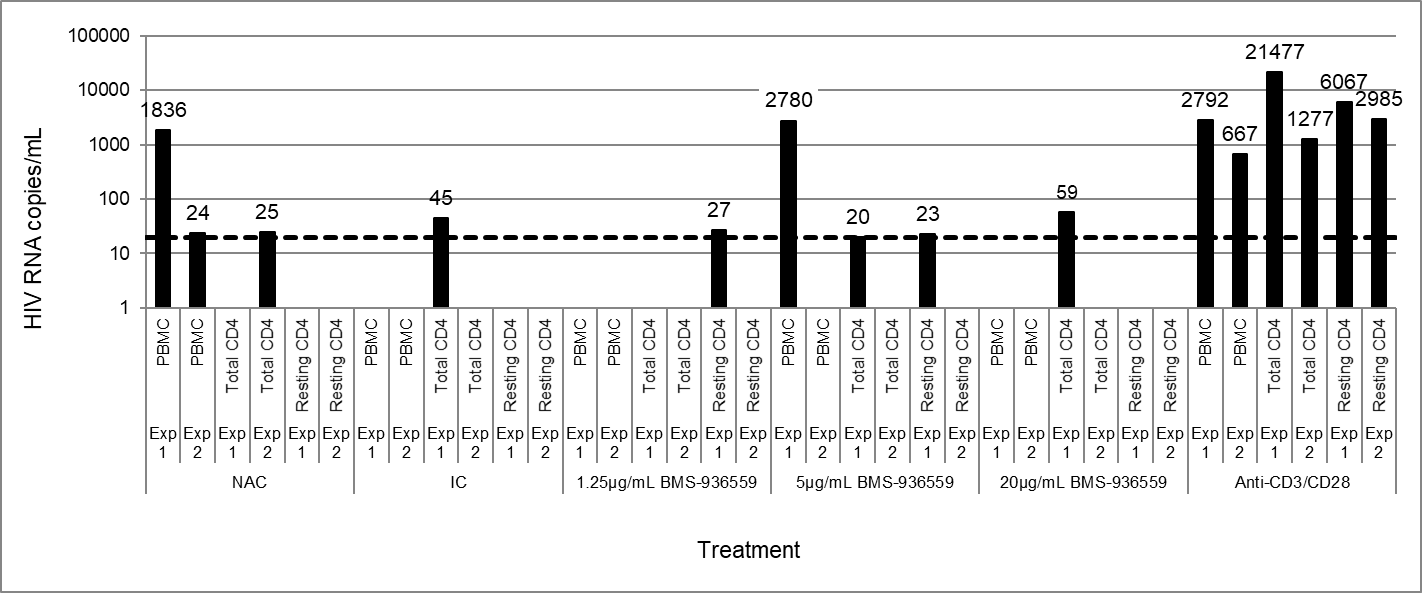
**

**B**

**
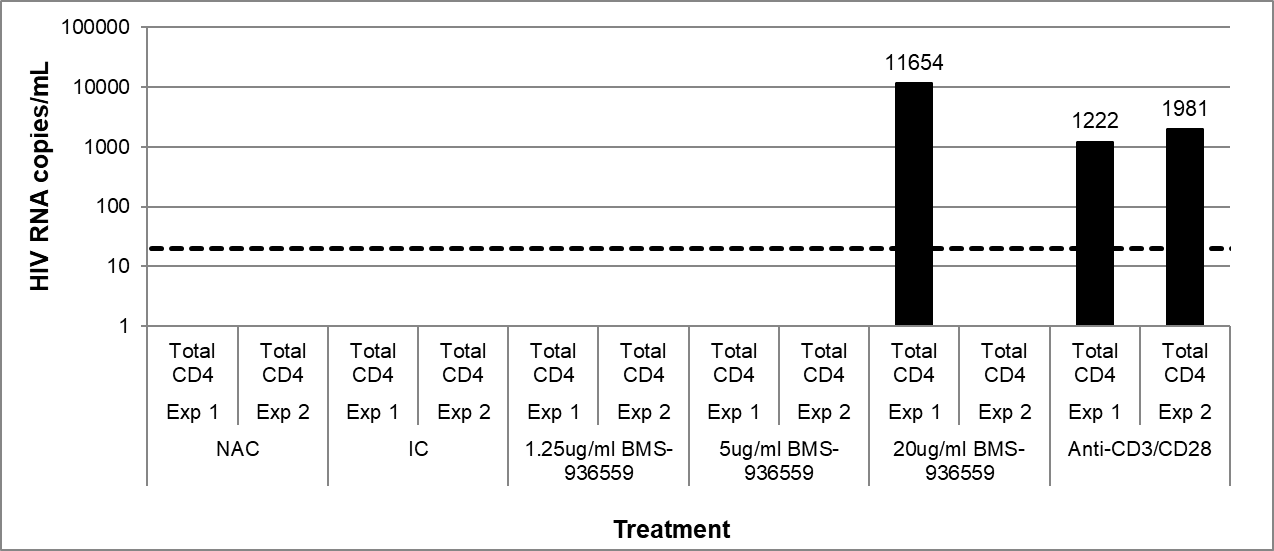
**

**C**

**
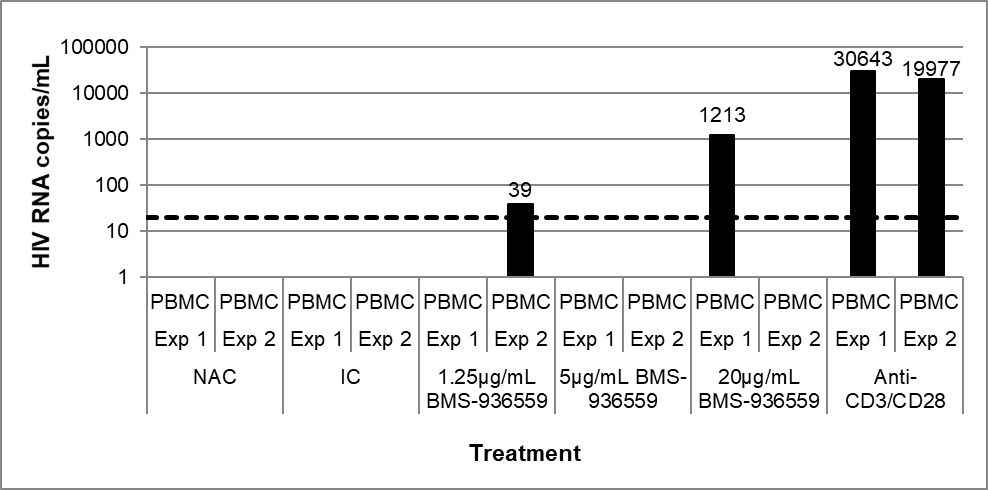
**

**D**

**
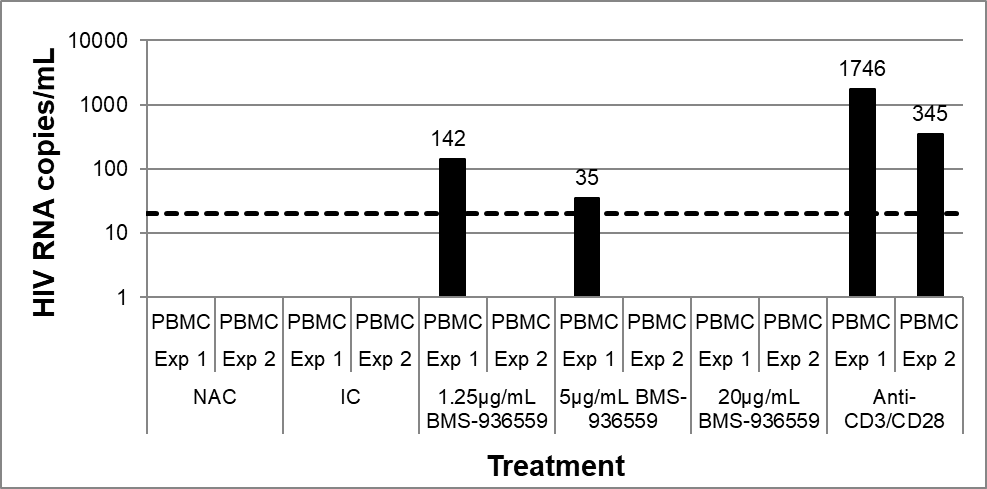
**

**E**

**
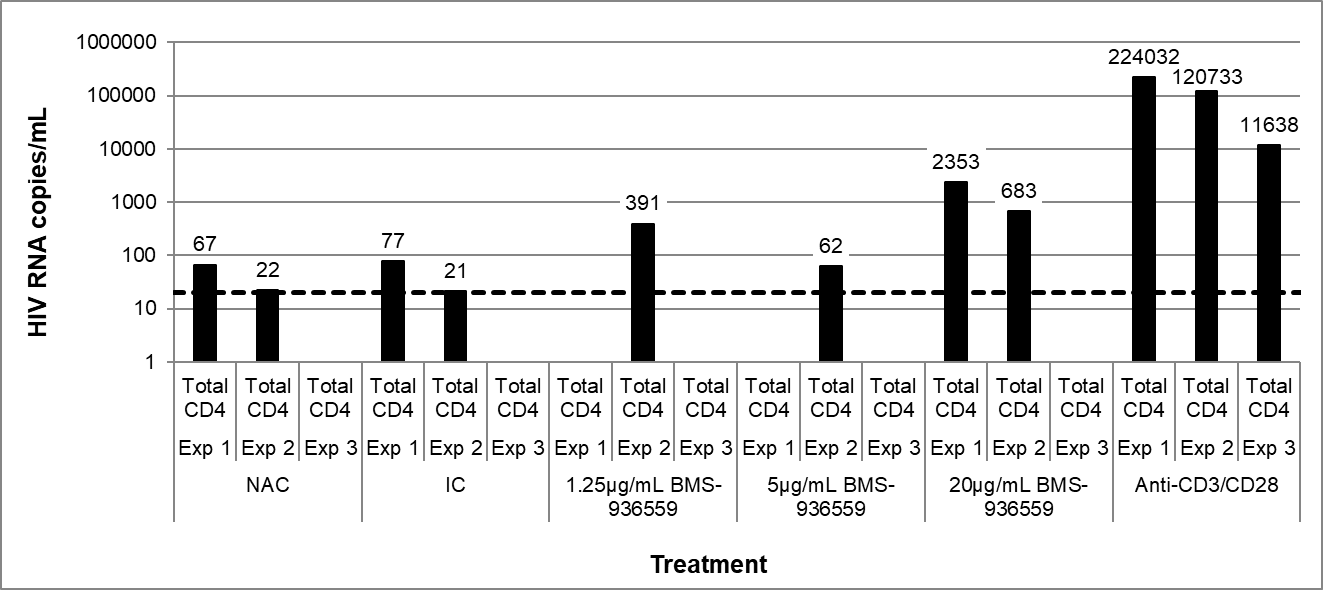
**
