# Supplementary material for: Blockade of the PD-1 axis alone is not sufficient to activate HIV-1 virion production from CD4+ T cells of individuals on suppressive ART
Source: PLoS One. 2019 Jan 25;14(1):e0211112. doi: 10.1371/journal.pone.0211112 (PMC6347234; doi:10.1371/journal.pone.0211112)
Supplement: S2 Fig — This schematic illustrates how CD4+ and CD8+ (CD4-) T-cells were gated by flow cytometry to measure PD-1 and PD-L1 expression. (DOCX) [file pone.0211112.s002.docx]

**S2 Fig Flow Cytometry Gating Strategy**

*
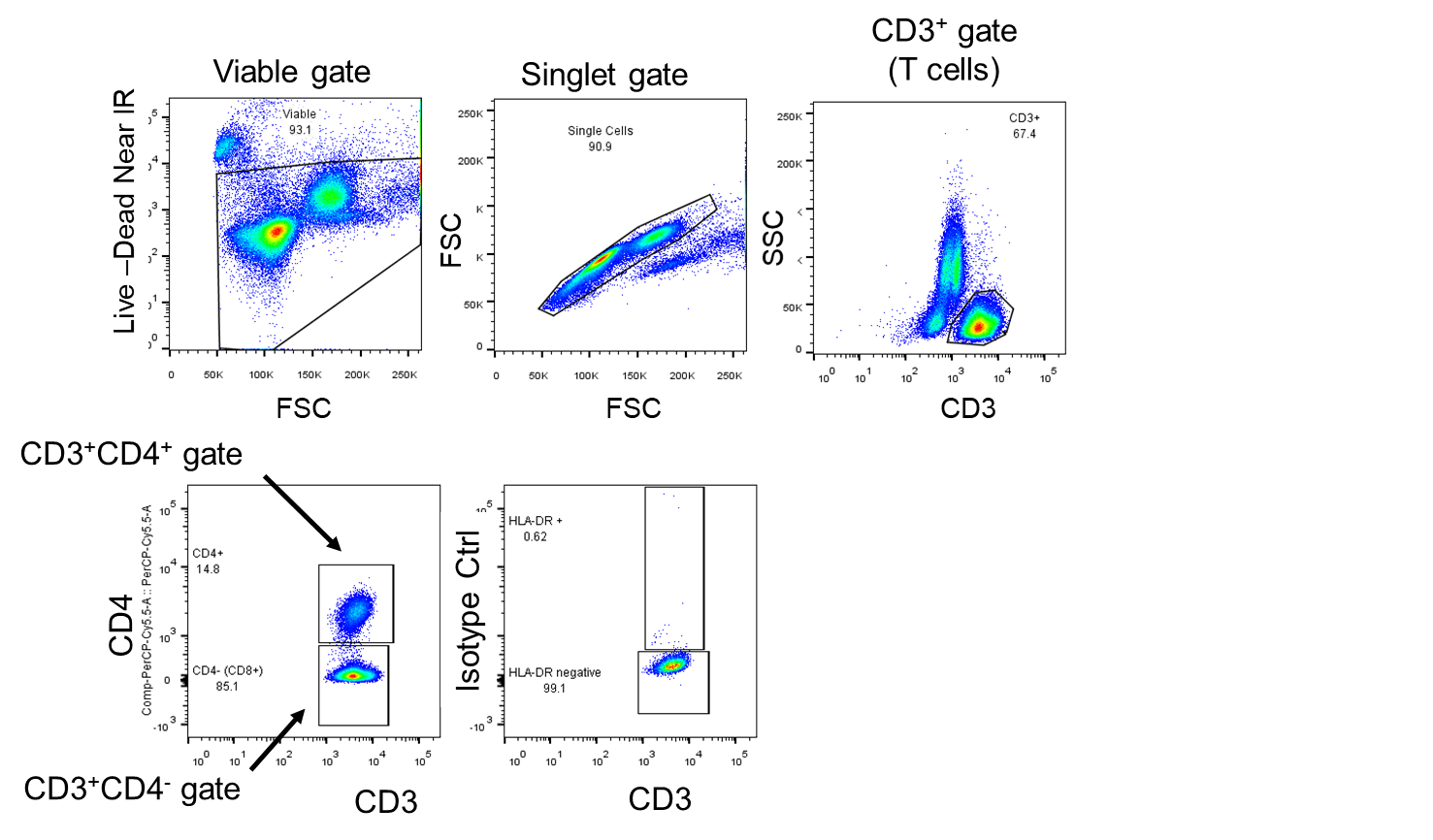
*

This schematic illustrates how CD4+ and CD8+ (CD4-) T-cells were gated by flow cytometry to measure PD-1 and PD-L1 expression.
