# Supplementary material for: Blockade of the PD-1 axis alone is not sufficient to activate HIV-1 virion production from CD4+ T cells of individuals on suppressive ART
Source: PLoS One. 2019 Jan 25;14(1):e0211112. doi: 10.1371/journal.pone.0211112 (PMC6347234; doi:10.1371/journal.pone.0211112)
Supplement: S1 Table — Virion production as HIV RNA copies/mL. Cells with yellow shading have virologic responses when defined as being greater than twice the virion production from cells treated with isotype control or > 60 copies/mL. Cells with bolded font have virologic responses when defined as being greater than three times the virion production from cells treated with isotype control or = > 90 copies/mL. BMS = BMS-936559, IC = isotype control, AC = activation control with anti-CD3/28, TND = HIV-1 RNA target not detected. (DOCX) [file pone.0211112.s003.docx]

**S1 Table Virion production in response to BMS-936559**


Virion production as HIV RNA copies/mL. Cells with yellow shading have virologic responses when defined as being greater than twice the virion production from cells treated with isotype control or > 60 copies/mL. Cells with bolded font have virologic responses when defined as being greater than three times the virion production from cells treated with isotype control or = > 90 copies/mL. BMS = BMS-936559, IC = isotype control, AC = activation control with anti-CD3/28, TND = HIV-1 RNA target not detected.

|  |  | B3 | W7 | E4 | E5 | T1 | A3 | C5 | F6 | K5 | M1 | R11 |
| --- | --- | --- | --- | --- | --- | --- | --- | --- | --- | --- | --- | --- |
| PBMC | MC | 1836 | TND | TND | TND | TND | TND | TND | < 20 | TND | < 20 | TND |
|  | IC | TND | TND | TND | TND | TND | TND | TND | TND | < 20 | TND | < 20 |
|  | 1.25 μg/mL BMS | TND | < 20 | TND | **142** | TND | TND | TND | < 20 | TND | TND | < 20 |
|  | 5 μg/mL BMS | **2780** | TND | **199** | 35 | < 20 | TND | TND | < 20 | < 20 | TND | TND |
|  | 20 μg/mL BMS | TND | **1213** | TND | < 20 | TND | TND | TND | TND | TND | TND | TND |
|  | AC | 2792 | 30643 | 8217 | 1746 | 581 | 40 | 105 | 3181 | 80889 | 1380 | TND |
| Total CD4^+^ T-cells | MC | < 20 | < 20 | 60 | < 20 |  | < 20 | < 20 | 36 | 67 | 80 | TND |
|  | IC | 45 | < 20 | TND | < 20 |  | < 20 | < 20 | 25 | 77 | 140 | < 20 |
|  | 1.25 μg/mL BMS | < 20 | 34 | 81 | 72 |  | < 20 | TND | < 20 |  | 74 | 47 |
|  | 5 μg/mL BMS | 20 | < 20 | 76 | < 20 |  | 33 | < 20 | 38 |  | 117 | < 20 |
|  | 20 μg/mL BMS | 59 | < 20 | 50 | < 20 |  | < 20 | **11654** | 37 | **2353** | 275 | 38 |
|  | AC | 21477 | 19224 | 26490 | 4143 |  | 801 | 1222 | 34255 | 224032 | 18322 | 5659 |
| Resting CD4^+^ T-cells | MC | < 20 | < 20 |  | < 20 |  | 69 |  | 37 | 119 | 103 | 20 |
|  | IC | < 20 | 26 |  | TND |  | TND |  | TND | 68 | 97 | 22 |
|  | 1.25 μg/mL BMS | 27 | 74 |  | < 20 |  | 26 |  | 54 |  |  | 22 |
|  | 5 μg/mL BMS | 23 | < 20 |  | TND |  | < 20 |  | TND |  | 52 | 48 |
|  | 20 μg/mL BMS | TND | < 20 |  | < 20 |  | < 20 |  | 33 | TND | 118 | < 20 |
|  | AC | 6067 | 550 |  | 396 |  | 823.5 |  | 27284 | 947.8 | 10924 | 39 |
