# Supplementary material for: Blockade of the PD-1 axis alone is not sufficient to activate HIV-1 virion production from CD4+ T cells of individuals on suppressive ART
Source: PLoS One. 2019 Jan 25;14(1):e0211112. doi: 10.1371/journal.pone.0211112 (PMC6347234; doi:10.1371/journal.pone.0211112)
Supplement: S2 Table — Virion production as HIV RNA copies/mL. 3/28 = anti-CD3/28, IC = isotype control, BMS = BMS-936559, TND = target not detected. (DOCX) [file pone.0211112.s004.docx]

**S2 Table Virion production in cells stimulated with anti-CD3/CD28 antibodies and BMS-936559**

Virion production as HIV RNA copies/mL. 3/28 = anti-CD3/28, IC = isotype control, BMS = BMS-936559, TND = target not detected.

|  |  | W7 | C5 experiment 1 | C5 experiment 2 | K5 |
| --- | --- | --- | --- | --- | --- |
| PBMC | 3/28 alone | 30643 | 105 |  |  |
|  | 3/28 + IC | 4343 | 1916 |  |  |
|  | 3/28+1.25μg/mL BMS | 11023 | 106 |  |  |
|  | 3/28+5μg/mL BMS | 19725 | 34 |  |  |
|  | 3/28+20μg/mL BMS | 12644 | 478 |  |  |
| Total CD4+ T-cells | 3/28 alone | 19224 | 1222 | 1981 | 11638 |
|  | 3/28 + IC | 13027 | 612 | 462 | 8559 |
|  | 3/28+1.25μg/mL BMS | 2493 | 2466 | 333 | 10371 |
|  | 3/28+5μg/mL BMS | 15279 | 3235 | 2456 | 5630 |
|  | 3/28+20μg/mL BMS | 13241 | 15224 | 2969 | 1088 |
| Resting CD4^+^ T-cells | 3/28 alone | 550 |  |  |  |
|  | 3/28 + IC | 378 |  |  |  |
|  | 3/28+1.25μg/mL BMS | 12695 |  |  |  |
|  | 3/28+5μg/mL BMS | 436 |  |  |  |
|  | 3/28+20μg/mL BMS | 743 |  |  |  |
