# Supplementary material for: Blockade of the PD-1 axis alone is not sufficient to activate HIV-1 virion production from CD4+ T cells of individuals on suppressive ART
Source: PLoS One. 2019 Jan 25;14(1):e0211112. doi: 10.1371/journal.pone.0211112 (PMC6347234; doi:10.1371/journal.pone.0211112)
Supplement: S3 Table — Virion production as HIV RNA copies/mL. Cells with yellow background have virologic responses when defined as being greater than twice the virion production from cells treated with isotype control or as > 60 copies/mL. Cells with bolded font have virologic responses when defined as being greater than three times the virion production from cells treated with isotype control or as > 90 copies/mL. IC = isotype control, nivo = nivolumab, AC = activation control with anti-CD3/28, TND = target not detected. (DOCX) [file pone.0211112.s005.docx]

**S3 Table Virion production in response to nivolumab**


Virion production as HIV RNA copies/mL. Cells with yellow background have virologic responses when defined as being greater than twice the virion production from cells treated with isotype control or as > 60 copies/mL. Cells with bolded font have virologic responses when defined as being greater than three times the virion production from cells treated with isotype control or as > 90 copies/mL. IC = isotype control, nivo = nivolumab, AC = activation control with anti-CD3/28, TND = target not detected.

|  |  | B3 | W7 | E4 | E5 | O5 | E1 | K4 | T1 | W1 |
| --- | --- | --- | --- | --- | --- | --- | --- | --- | --- | --- |
| PBMC | MC | TND | TND | < 20 | < 20 | < 20 | TND | 38 | LOD | 68 |
|  | IC | TND | TND | < 20 | < 20 | TND | < 20 | 131 | LOD | 87 |
|  | 5 μg/mL nivo | TND | TND | TND | TND | TND | TND | LOD | LOD | 38 |
|  | 20 μg/mL nivo | TND | < 20 | **137** | TND | TND | TND | 332 | LOD | LOD |
|  | AC | 181 | 3073 | 6147 | 728 | 45 | 62 | 957 | 132 | 4428 |
|  | AC + IC | 433 | 305 | 506 | 1002 | 107 | 105 | 1383 | 165 | 7023 |
|  | AC + 5 μg/mL nivo | 322 | 333 | 1235 | 1409 | 248 | TND | 770 | 155 | 1214 |
|  | AC + 20 μg/mL nivo | 3389 | 14275 | 1250 | 122 | 33 | 299 | 357 | 120 | 173 |
| CD8-depleted PBMC | MC |  |  | TND | TND | TND | TND | LOD | LOD | LOD |
|  | IC |  |  | TND | TND | TND | TND | 42 | LOD | LOD |
|  | 5 μg/mL nivo |  |  | **1994** | < 20 | TND | < 20 | 63 | LOD | 78 |
|  | 20 μg/mL nivo |  |  | TND | TND | TND | TND | **198** | LOD | **299** |
|  | AC |  |  | 28571 | 2907 | 107 | 1184 | 2970 | 926 | 8601 |
|  | AC + IC |  |  | 20598 | 7605 | 168 | < 20 | 1425 | 35 | 233 |
|  | AC + 5 μg/mL nivo |  |  | 10185 | 1382 | 897 | 2439 | 2136 | 60 | 4970 |
|  | AC + 20 μg/mL nivo |  |  | 25904 | 6771 | 146 | 41 | 6687 | 72 | 3638 |
| Total CD4^+^ T-cells | MC | TND | < 20 | 44 | 65 | TND | TND | LOD | LOD | LOD |
|  | IC | < 20 | < 20 | < 20 | TND | TND | 51 | LOD | LOD | 56 |
|  | 5 μg/mL nivo | TND | TND | 38 | TND | < 20 | < 20 | **117** | LOD | **1517** |
|  | 20 μg/mL nivo | < 20 | 35 | < 20 | < 20 | TND | < 20 | **185** | LOD | 54 |
|  | AC | 5098 | 5310 | 20751 | 18726 | 10233 | 6752 | 29006 | 2480 | 23933 |
|  | AC + IC | 68240 | 5665 | 37736 | 11943 | 512 | 4260 | 14517 |  | 12657 |
|  | AC + 5 μg/mL nivo | 10126 | 16060 | 15926 | 31406 | 89 | 1127 | 23001 |  | 38112 |
|  | AC + 20 μg/mL nivo | 8131 | 2036 | 23616 | 13397 | 4314 | 1485 | 29682 |  | 19655 |
