# Supplementary material for: Blockade of the PD-1 axis alone is not sufficient to activate HIV-1 virion production from CD4+ T cells of individuals on suppressive ART
Source: PLoS One. 2019 Jan 25;14(1):e0211112. doi: 10.1371/journal.pone.0211112 (PMC6347234; doi:10.1371/journal.pone.0211112)
Supplement: S4 Table — The proportion of cells expressing PD-1 or PD-L1 were measured by flow cytometry on CD4+ T-cells and CD8+ T-cells. N/A = Not Applicable. (DOCX) [file pone.0211112.s006.docx]

**S4 Table PD-1 and PD-L1 expression by flow cytometry**.

The proportion of cells expressing PD-1 or PD-L1 were measured by flow cytometry on CD4+ T-cells and CD8+ T-cells.

N/A = Not Applicable.


| Donor ID | Assay | % PD1+ of CD8+ CD3+ gate | % PD1+ of CD4+ CD3+ gate | % PDL1+ of CD8+ CD3+ gate | % PDL1+ of CD4+ CD3+ gate | Viral response by 2x control | Viral response by 3x control |
| --- | --- | --- | --- | --- | --- | --- | --- |
| K4 | Nivolumab | 14.8 | 13.7 | 1.6 | 2.6 | yes | yes |
| W1 | Nivolumab | 11.7 | 26.2 | 2.8 | 5.8 | yes | yes |
| E4 | Nivolumab | 30.1 | 34.1 | 3.6 | 13.7 | yes | yes |
| O5 | Nivolumab | 25.5 | 35.2 | 5.1 | 11.1 | no | no |
| E5 | Nivolumab | 25.3 | 43.3 | 15 | 33.8 | no | no |
| E1 | Nivolumab | 24.7 | 41.1 | 5.3 | 15.9 | no | no |
| T1 | Nivolumab | 11.7 | 54 | 9.1 | 20.9 | no | no |
| W7 | Nivolumab | 25.5 | 37.3 | 24 | 42.7 | no | no |
| B3 | Nivolumab | 22.8 | 31.3 | 17 | 39 | no | no |
| W7 | BMS-936559 | 20.8 | 34.2 | 8.7 | 18.5 | yes | yes |
| R1 | BMS-936559 | 31.8 | 50.7 | 31.2 | 25.6 | yes | no |
| E5 | BMS-936559 | 29.9 | 38.5 | 49.7 | 60.6 | yes | yes |
| K5 | BMS-936559 | 42.5 | 50.7 | 41.2 | 52.4 | yes | yes |
| B3 | BMS-936559 | 19.3 | 28.4 | 7.2 | 16.9 | yes | yes |
| A3 | BMS-936559 | 41.4 | 66.4 | 16.8 | 36.2 | no | no |
| F6 | BMS-936559 | 19.6 | 35.8 | 41.4 | 44.8 | no | no |
| M1 | BMS-936559 | 32.2 | 55.5 | 37.9 | 50.8 | no | no |
| E4 | BMS-936559 | 29.3 | 46.1 | 9.7 | 31.1 | yes | yes |
| C5 | BMS-936559 | 30.7 | 26.0 | 26.7 | 24.1 | yes | yes |
| T1 | BMS-936559 | 23.0 | 51.3 | 13.6 | 21.6 | no | no |
| Healthy Donor 1 | N/A | 32.4 | 25.5 | 13.0 | 20.5 | N/A | N/A |
| Healthy Donor 2 | N/A | 44.9 | 31.0 | 30.4 | 35.6 | N/A | N/A |
| Healthy Donor 3 | N/A | 34.8 | 40.3 | 3.64 | 11.38 | N/A | N/A |
